# Supplementary material for: Bayesian Inference of Infectious Disease Transmission from Whole-Genome Sequence Data
Source: Mol Biol Evol. 2014 Apr 8;31(7):1869–79. doi: 10.1093/molbev/msu121 (PMC4069612; doi:10.1093/molbev/msu121)
Supplement: Supplementary Data [file supp_31_7_1869__index.html]

Bayesian inference of infectious disease transmission from whole genome sequence data — Bayesian Inference of Infectious Disease Transmission from Whole-Genome Sequence Data — Bayesian Inference of Infectious Disease Transmission from Whole-Genome Sequence Data — Supplementary Data 

# Bayesian Inference of Infectious Disease Transmission from Whole-Genome Sequence Data

## Supplementary Data

files

**Files in this Data Supplement:**

- Supplementary Data - pdf file
