# Supplementary material for: Bayesian Inference of Infectious Disease Transmission from Whole-Genome Sequence Data
Source: Mol Biol Evol. 2014 Apr 8;31(7):1869–79. doi: 10.1093/molbev/msu121 (PMC4069612; doi:10.1093/molbev/msu121)
Supplement: Supplementary Data [file supp_msu121_supplement.pdf]

## Supplementary Information

In the main text we described the moves in the MCMC: to update the transmission tree $T$, we choose a single infection event at random. If the infection is to the source case, then its date is modified by a draw from Uniform($[-\epsilon, \epsilon]$) (excluding values that would make the transmission to the source case become more recent than the common ancestor of the genealogy $G$). If the transmission is not to the first case, then it corresponds to a transmission event, ie a point at which two colours meet on the genealogy. In this case, the MCMC step consists of moving this transmission event uniformly at random to another point on the genealogy where it gives a valid colouring. This point must have the properties that in the resulting colouring: (i) there are only $n-1$ colour changes, (ii) each leaf is coloured in the colour $c_i$ of the host it corresponds to and (iii) the colour $c_i$ does not exist in the tree after the leaf corresponding to host $i$.

It is clear that moves involving transmission to the source case are symmetric. To see that other proposed moves are also symmetric, consider removing a transmission event $c$ (say from A to B). This leaves an invalid tree colouring, because there will no longer be any point separating A and B, so their tips will have the same colour, violating (ii) above. A valid colouring can be obtained by re-inserting a transmission event between A and B somewhere along the A-B path. However, only a part of this path will be a valid destination for the point to be inserted; some parts of the path will be after the recovery of A or B, and some choices may result in changes in the direction of transmission for other points in the colouring such that *they* do not meet condition (iii) above. But there is a part, $P_{ab}$, of the A-B path where it *is* possible to re-insert the point ($P_{ab}$ includes at least a small interval surrounding the initial point $c$) , and in the MCMC move, we re-insert the point with uniform probability on $P_{ab}$. Call the new point $c'$. If we remove $c'$, we are left with the same part $P_{ab}$ of the A-B path on which we can re-insert it with uniform probability. So the probability of moving from $c$ to $c'$ is the same as the probability of moving from $c'$ to $c$.

The Markov chain is irreducible if any valid colouring (ie any transmission tree $T$ consistent with the genealogy $G$) can be reached with positive probability from any starting colouring through a sequence of the described moves. Consider the transmission tree (and associated colouring) $A^*$ in which one host, $A$, is infected first and subsequently infects all other hosts. While it may be the case that some hosts could not have infected all others, because they recover before the rest of the genealogy has branched sufficiently, there are always at least two hosts who could be chosen as $A$. These are the hosts whose recovery times are later than all of the internal branching events in the tree. The fact that $G$ is a phylogeny (ie a full, rooted binary tree) guarantees that there are at least two such hosts, namely the hosts corresponding to the two tips descending from the latest internal node in $G$. In the colouring $A^*$, host $A$'s colour extends up the tree from $A$ to the root, and down each lineage in the tree, changing colour to each host $j$'s colour somewhere on $j$'s pendant edge (for all $j \neq A$). Because $A$ recovered after every internal node in the tree, this colouring meets condition (iii), and it is clear that it meets conditions (i) and (ii) as well. So $A^*$ is a valid colouring.

We proceed by induction. On the 2-leaf tree with tips $A$ and $B$, we must have either $A$ or $B$ infected initially, and at some time, one infects the other. By moving the (unique) transmission point sufficiently far along the path to $B$ that it is on $B$'s side of the root, we obtain the colouring $A^*$ for this tree. Now, assume that $A^*$ can be reached for any coloured tree with $k$ tips.

Consider a coloured tree, $T_{k+1}$ with $k+1$ tips. All outbreaks contain at least one host who did not infect any others hosts in the sample, or at least did not infect any other hosts with a lineage in the sample. If not, there would be at least $n+1$ infections including the source of the outbreak, and only $n$ hosts, so by the pigeonhole principle, at least one host would receive more than one infection, contradicting the SIR model[1].

Let such a host be $B$. Because $B$ did not infect any other hosts, the pendant edge from tip $B$ to its most recent ancestor changes hosts at some time $t_B$ prior to its coalescence with its most recent ancestor (otherwise, $B$'s colour would transition forward in time to $C$'s colour for some $C$, meaning $B$ infected $C$). So $B$'s colour changes on the pendant edges to $B$. First, assume that $B \neq A$. Now remove $B$ and its pendant edge $e_B$ from the tree. By supposition the remaining tree has $k$ leaves, and therefore a sequence of moves can be found that reaches the colouring $A^*$. Now replace $B$ and its pendant edge, with $B$'s colour assigned to edge $e_B$ after time $t_B$, and $A$'s colour assigned to edge $e_B$ prior to $t_B$. This is the colouring $A^*$ on $T_{k+1}$.

It remains to discuss the case $B = A$. Again, remove $B$, but now set $A'$ to be the other host who recovered after all of the internal branching events in $T_{k+1}$. By assumption, we can reach the colouring $A'^*$ through a sequence of MCMC moves, because removing $B$ leaves a tree with only $k$ tips. Now re-attach $B$, placing a colour change $c$ from $A'$'s colour to $B$'s, on $B$'s pendant edge. This is the colouring $A'^*$ on $T_{k+1}$, so all hosts have colour changes on their pendant edges except $A'$. Now, because $B$ and $A'$ are the two hosts who descend from the latest internal node of the tree, the path from $A'$ to $B$ consists of the union of their pendant edges. Moving $c$ from $B$'s pendant edge to $A'$'s is a valid move, because it doesn't affect the number of colours (condition (i) is met), it does not change the colour assigned to any leaf (condition (ii) is met), and it does not require that any colour be present in the tree after the associated tip's recovery time, satisfying condition (iii). Moving $c$ to $A'$'s pendant edge yields the colouring $A^*$, and this completes the inductive argument that colouring $A^*$ can always be reached through a sequence of moves, so the Markov chain is irreducible.

[1]Multiple infections per host (re-infection; mixed infection) are consistent with our approach, but would require multiple isolates per host. In this case, hosts with more than one distinct infection would correspond to more than one tip in the genealogy. The same framework would apply, but the wording would need to be somewhat altered; each colour in the tree would correspond to an isolate, not necessarily to a host, and each isolate would have at most one other lineage in the tree that was its ancestor in the outbreak. As such, our work does not assume that re-infection and multiple infection do not happen, but if these are to be included, then each separate infection would correspond to its own tip and its own colour in the genealogy.

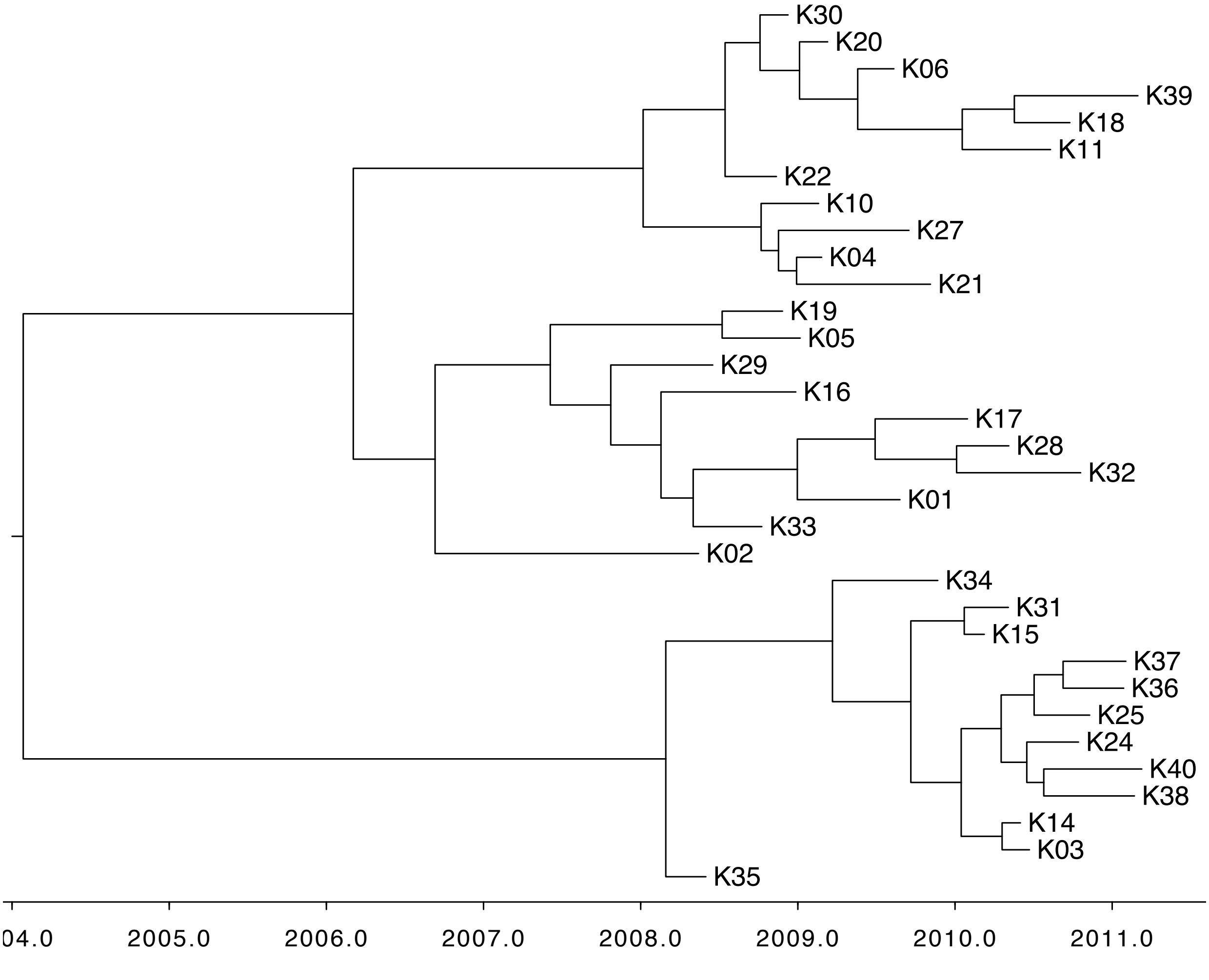


**Figure S1.** Maximum credibility consensus tree computed by BEAST for the tuberculosis dataset.

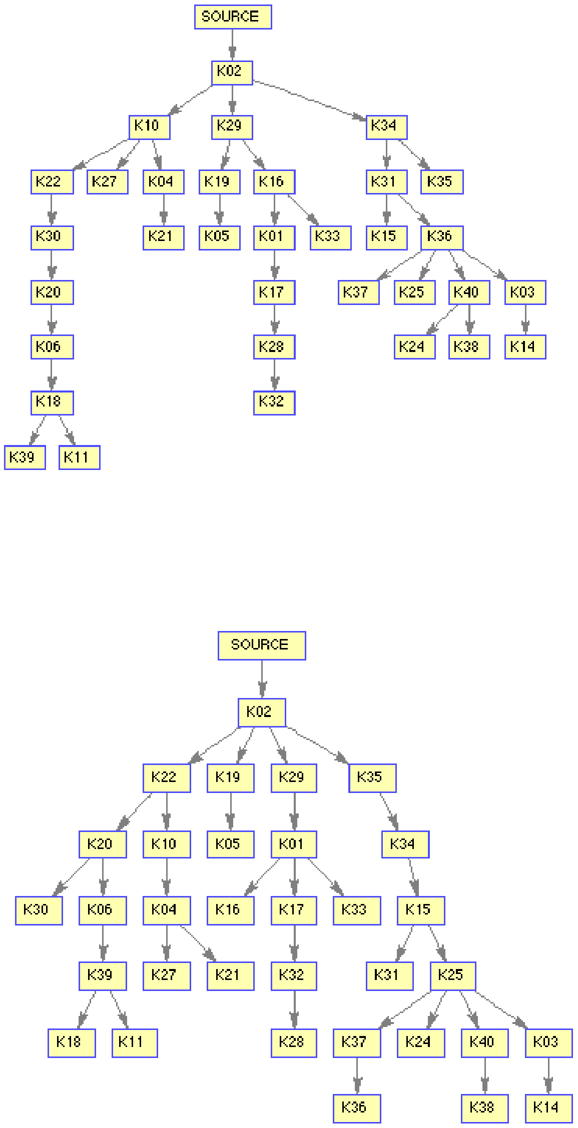


**Figure S2.** Posterior modes of the transmission tree without (top) and with (bottom) additional epidemiological data.

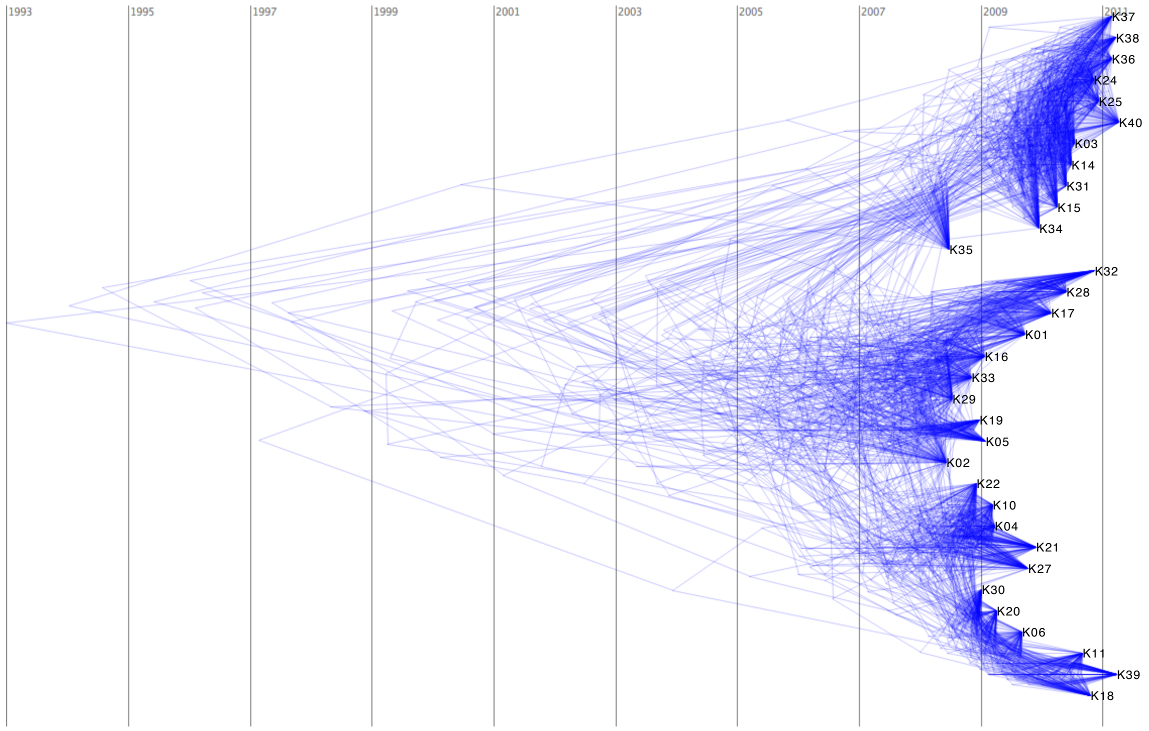


**Figure S3.** Posterior sample of 100 phylogenetic trees computed by BEAST for the tuberculosis dataset.

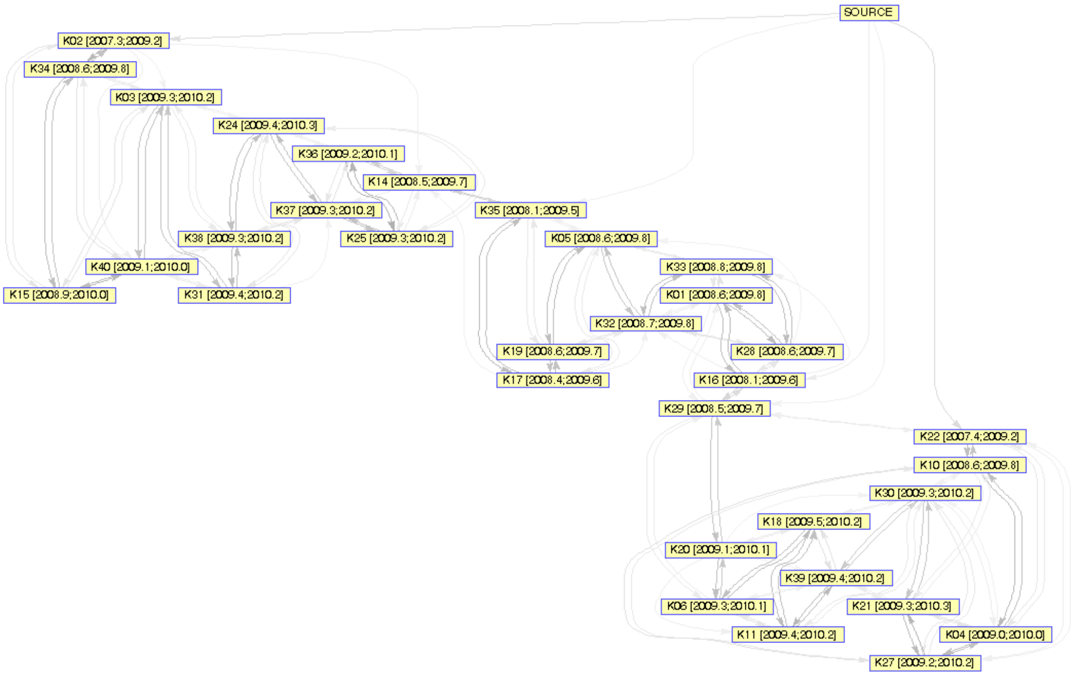


**Figure S4.** Application to the tuberculosis outbreak based on the 100 phylogenetic trees shown in Figure S3. No additional epidemiological information was used. Edges shown in the posterior transmission tree have a probability greater than 5%.

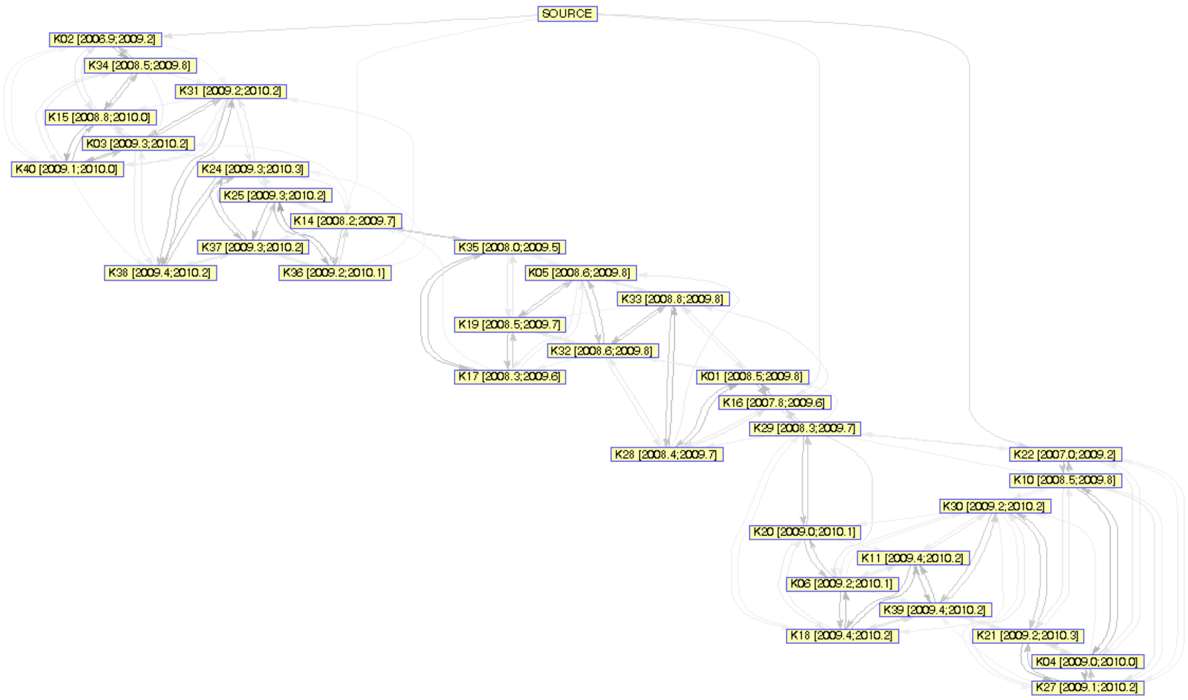


**Figure S5.** Application to the tuberculosis outbreak based on the 100 phylogenetic trees shown in Figure S3. Additional epidemiological information was used. Edges shown in the posterior transmission tree have a probability greater than 5%.
